# Antibody Therapies in Autoimmune Inflammatory Myopathies: Promising Treatment Options

**DOI:** 10.1007/s13311-022-01220-z

**Published:** 2022-04-08

**Authors:** Rachel Zeng, Stefanie Glaubitz, Jens Schmidt

**Affiliations:** 1 Muscle Immunobiology Group, Neuromuscular Center, Department of Neurology, University Medical Center Göttingen, Göttingen, Germany; 2 Department of Neurology and Pain Treatment, Immanuel Klinik Rüdersdorf, University Hospital of the Brandenburg Medical School Theodor Fontane, Rüdersdorf bei Berlin, Germany; 3 Faculty of Health Sciences Brandenburg, Brandenburg Medical School Theodor Fontane, Rüdersdorf bei Berlin, Germany

**Keywords:** Myositis, Treatment, Inflammatory myopathies, Antibodies, Clinical trial, Rituximab

## Abstract

Inflammatory myopathies, including polymyositis (PM), dermatomyositis (DM), inclusion body myositis (IBM), necrotizing myopathy (NM), antisynthetase syndrome (ASS) and overlap myositis (OM), in short myositis, are rare diseases. All forms of myositis have progressive muscle weakness in common, with each subtype characterized by different autoantibody profiles, histological findings and extramuscular manifestations. Due to better understanding of the pathogenesis of the muscle inflammation in myositis, new molecular pathways for targeted therapy have been discovered. Current therapies aim at different components of the innate or the adaptive immune response. Additionally, non-inflammatory mechanisms in myositis have come into focus as possible treatment targets. The use of therapeutical antibodies in myositis has been examined in various clinical studies, several of them randomized controlled ones: Depletion of B-cells by rituximab has been established as treatment of refractory myositis. IVIG, an antibody therapy in the wider sense, has now been licensed for DM following a recent positive clinical trial. Negative study results were reported in randomized trials with infliximab, sifalimumab and bimagrumab. Studies on basiliximab and eculizumab are currently underway, and are expected to yield results in a couple of years. Despite some promising results of clinical studies with antibody therapy in myositis, further research is crucial to optimize the treatment for this debilitating disease and to find treatment alternatives for treatment-refractory patients.

**Supplementary Information:**

The online version contains supplementary material available at 10.1007/s13311-022-01220-z.

## Introduction

Inflammatory myopathies, generally called myositis, are a group of heterogeneous diseases including the subtypes dermatomyositis (DM), juvenile dermatomyositis (JDM), polymyositis (PM), necrotizing myopathy (NM), antisynthetase syndrome (ASS), overlap myositis (OM) and inclusion body myositis (IBM).

The myositis subtypes are all rare diseases. The incidence ranges from 2 to 5 cases per million in 1 year. Whereas IBM affects more men than women and occurs in the older age, the other subtypes more commonly affect middle-aged women [1]. JDM also occurs more in girls; the mean age of manifestation is around 6.7 years in girls and 7.3 years in boys [2].

The most recent classification for myositis is the 2017 EULAR/ACR criteria for adult and juvenile idiopathic inflammatory myopathies, which use clinical, serological and histological parameters to identify the most common myositis subtypes DM, JDM, PM and IBM [3]. The common symptom in all myositis subtypes is subacute or chronic muscle weakness caused by autoimmune damage of the muscles, resulting in physical disability. Additional typical clinical findings in DM and JDM are skin lesions and calcinosis, which can be the leading symptoms. Patients can suffer from interstitial lung disease and arthralgia, especially with an ASS. Besides the involvement of lungs and joints, all myositis subtypes can show further extramuscular manifestations of the myocardium and kidney, as well as dysphagia due to involvement of the pharyngeal muscles [4].

Laboratory tests usually show an increase of creatine kinase, and frequently the presence of myositis-specific and/or myositis-associated autoantibodies. They can aid in differentiating the subtypes and estimating the risk for extramuscular involvement and neoplasia [4]. For instance, patients with anti-MDA5 antibodies are at higher risk for severe lung involvement [5], whereas the occurrence of anti-TIF1-γ and anti-NXP2 antibodies is highly associated with malignancy [6].

A further important diagnostic tool is a muscle biopsy with comprehensive histological evaluation. Muscle biopsies show characteristic histopathological patterns of inflammatory mechanisms. In DM, the muscle biopsy shows complement activation and intravascular deposition of the membrane attack complexes (MAC), which lead to capillary destruction, necrosis of muscles fibres and perivascular inflammation with CD4+ T-cells [7, 8]. In PM and IBM, CD8+ T-cells dominating the infiltrate and MHC-I upregulation on muscle fibres are typical findings. Moreover, IBM is characterized by inclusion bodies consisting of amyloid and other degenerative protein aggregates [9].

So far, the aetiology of myositis is complex and not completely understood. Similar to other autoimmune diseases, the development of myositis is assumed to result from an interaction of various factors. On the one hand, genetic risk factors such as the HLA 8.1 ancestral haplotype and other genetic variants have been identified [10, 11]. Additionally, a variety of environmental factors have been proposed to be associated with an increased risk of myositis, possibly acting on top of genetic susceptibility. These include certain viral or bacterial infections, smoking, medications and ultraviolet radiation, although evidence is mainly based upon animal models, case reports or case series [12].

The disease mechanisms that lead to muscle inflammation and damage involve the innate immune system, including cytokines and chemokines, as well as the adaptive immune system, including the specific B- and T-cell response. In addition, there is growing evidence of the importance of non-immune-mediated factors, such as cell stress and mitochondrial damage, as well as impaired autophagy [12]. In order to develop possible targets for therapies in myositis, research interest focuses on the various pathomechanisms and molecular key players in myositis. Due to increasing understanding of the pathogenesis in the different subtypes of myositis, new specific targets have been discovered and an individualized therapy becomes more feasible.

**Fig. 1:**
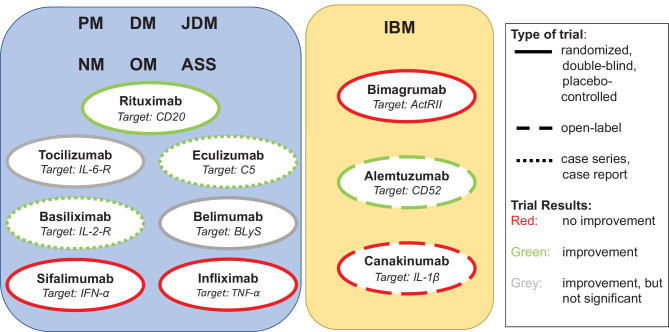
Overview of antibody therapies tested in myositis. The figure provides an overview of antibody therapies that have been tested for efficacy and tolerability in myositis to date. The left panel includes antibody therapies that have been tested in polymyositis (PM), dermatomyositis (DM), juvenile dermatomyositis (JDM), necrotizing myopathy (NM), overlap myositis (OM) and antisynthetase syndrome (ASS). The right panel shows antibody therapies that have been tested in inclusion body myositis (IBM). The colours illustrate the study outcomes: Green-framed study drugs have shown improvement in clinical trials, red-framed study drugs achieved no improvement in clinical trials and grey-framed study drugs achieved some improvements, but did not reach statistical significance. Solid lines display randomized, double-blind and placebo-controlled clinical trials. Dashed lines point out open-label trials, and dotted lines indicate case series and case reports. Several antibody therapies have been examined in randomized, double-blind and placebo-controlled clinical trials. For basiliximab and eculizumab, only case series or case reports are available so far. Several trials have shown beneficial treatment effects in myositis (green)

The following sections summarize the evidence existing for antibody therapy in myositis, from well-established agents to new approaches and targeted therapies in myositis. Figure 1 provides an overview of antibody therapies in myositis to date.

## Current Treatment for Myositis

For control of disease activity, initial immunosuppression in myositis usually consists of high-dose glucocorticoids. To reduce side effects of glucocorticoids and for a more efficient long-term treatment, glucocorticoids are usually tapered and combined with other immunosuppressive agents like methotrexate, azathioprine or mycophenolate mofetil [13].

For non-responders or in case of severe extramuscular manifestations, second-line agents include cyclosporine and tacrolimus, which have shown beneficial effects in several studies in myositis patients [14]. Cyclophosphamide, another classical immunosuppressant, is reserved for treatment escalation in severe cases of myositis or systemic organ involvement, often in combination with other immunomodulatory agents, e.g. rituximab and IVIG [15]. Leflunomide, known in the treatment of rheumatoid arthritis, has been assessed as add-on therapy in refractory DM in a retrospective study, showing good efficacy especially for the control of cutaneous activity [16], but is currently not considered part of the standard treatment regime in myositis.

The beneficial effect of immunoglobulins (IVIg) in the treatment of refractory DM and PM has been proposed for decades, with the first double-blind, placebo-controlled, crossover study with 15 steroid-resistant DM patients published in 1993, demonstrating significant clinical and histological improvements on repeated muscle biopsies after 3 months of monthly IVIg infusions [17]. Multiple studies on the use of IVIg in myositis followed (reviewed in [18]), and IVIg has long been used off-label as add-on therapy for non-responders, being particularly useful in children or if immunosuppressants are not tolerated or may not be applicable, as during pregnancy, severe infections or neoplasia. Recently, a phase III, multicenter, double-blind, placebo-controlled, randomized trial evaluating the long-term efficacy and safety of IVIg in patients with DM has been completed (“ProDERM study”) (ClinicalTrials.gov Identifier: NCT02728752) ([19] journal publication of final results are expected soon). By reaching its primary endpoint as measured by the ACR/EULAR myositis response criteria at week 16, the successful study results led to approval of Octagam® by the FDA and EMA for the treatment of DM in adults. There is also positive evidence for the use of IVIg in patients with NM in some retrospective studies [20, 21] and a prospective, open-label trial in 6 NM patients [22]; however, larger randomized and controlled trials are still lacking.

Biologicals with various mechanisms of action and different targets in the immune response have been studied in myositis.

One example from the field of biologicals is abatacept. It is a fusion protein selectively blocking the interaction between antigen-presenting cells (APC) and T-lymphocytes by binding to CD80 and CD86 receptors on APC, resulting in a decreased activation and proliferation of T-cells. A phase IIb study showed beneficial effects on patients with refractory DM and PM [23] and has led to a subsequent phase III study (ClinicalTrials.gov Identifier: NCT02971683).

Another example are the Janus kinases (JAK) inhibitors, including tofacitinib, ruxolitinib and baricitinib. JAK play an important role in the interferon-meditated activation of cytokine receptors, leading to recruitment of the signal transducer and activator of transcription (STAT) factors, which modulate gene expression. Blocking the JAK–STAT pathway leads to decrease of interferons and interleukins. Several case series demonstrated a beneficial effect of JAK inhibitors in myositis [24, 25]. Efficacy was shown in a pilot study with tofacitinib in adult patients with DM regarding disease activity and skin manifestations [26]. Currently, a phase IIa trial with baricitinib (JAK 1/2 inhibitor) is ongoing (ClinicalTrials.gov Identifier: NCT04208464).

Treatment options for IBM differ from those of the other myositis subtypes. Currently, there is no effective therapy for patients with IBM. Studies with glucocorticoids as well as with biologicals and DMARD missed their primary endpoints [29]. The use of IVIg in IBM is a disputed issue, although there have been a few controlled trials. These include a double-blind, placebo-controlled trial with 19 IBM patients in 1997, in which a significant improvement of swallowing was noted [30]; later on, two other double-blind controlled studies were performed that showed only mild but insignificant improvements [31, 32]. Although all of the controlled trials have missed their primary endpoints, a central critique is that the applied treatment durations of 3 or 6 months might be too short to fully assess treatment response in the context of a slowly progressive disease. Therefore, and due to the lack of other treatment options, an exploratory use of IVIg can be justifiable in selected patients [33].

A promising approach in the therapy of IBM is sirolimus, an mTor inhibitor, which can decrease the proliferation of T-effector cells and preserves T-regulatory cells. Furthermore, it induces autophagy. Although the primary endpoint of a phase II trial was not formally met, therapy with sirolimus showed a positive effect on secondary endpoints such as walking ability [34]. A worldwide phase III trial is currently in preparation (ClinicalTrials.gov Identifier: NCT04789070).

In general, the therapeutic regime should be adjusted according to the disease activity and routinely monitored by appropriate disease activity scales and questionnaires as well as muscle force testing. The need for escalation or de-escalation has to be checked on a regular basis.

In addition to the pharmacological treatment, moderate physical training is a mainstay of the treatment and has been shown to improve muscle weakness in myositis [35].

In case of extramuscular manifestation, interdisciplinary patient care is essential. This interdisciplinary therapeutic team should consist of neurologists, rheumatologists, dermatologists, pathologists, pulmonologists and physical therapists to ensure optimal patient care.

## Antibody Therapies in DM, PM, ASS, OM and NM

### Rituximab

Rituximab, a monoclonal antibody directed against CD20 on B-cells, has been extensively studied in myositis refractory to standard treatment. Rituximab was effective in several case series of DM and PM, showing a steroid-sparing effect as well as improvement in muscle enzymes levels, muscle strength and lung function tests [36–39]. A large double-blind, randomized, placebo-phase trial on 195 patients with PM, adult DM and juvenile DM treated with rituximab (“RIM trial”) failed its primary endpoint, but showed significant clinical improvement and a steroid-sparing effect in 83% of the study participants by week 44 [27]. Furthermore, a significant improvement of refractory skin rashes in both adult and juvenile DM patients as assessed using cutaneous disease activity and damage scores was observed [40]. Therefore, the RIM trial is generally interpreted as a positive trial and has laid the grounds for the use of rituximab as a therapy escalation in refractory and severe cases of myositis. Post hoc analysis of the RIM trial demonstrated that patients with anti-Jo1 or anti-Mi-2 had a shorter time to treatment response and levels of these autoantibodies correlated with disease activity [41, 42].

The clinical relevance of the respective autoantibody during rituximab treatment was assessed in a registry-based study with 43 myositis patients, which showed that the majority of both antisynthetase antibody (ARS-ab)–positive and ARS-ab-negative patients had moderate or major improvements, but only the ARS-ab-positive group experienced a significant steroid-sparing effect [43].

Interstitial lung disease (ILD) represents a severe pulmonary complication in myositis and is common in ASS [44]. Rituximab has shown positive treatment effects in patients with ASS and ILD in that pulmonary function tests and radiographic signs on chest CT have improved in several retrospective studies [45–47] and one small prospective, open-label study [48]. A large, randomized controlled trial comparing the efficacy of rituximab versus cyclophosphamide in patients with ILD associated with connective tissue diseases, including myositis, is currently ongoing and will hopefully provide valuable evidence for the optimal treatment of this group of patients (RECITAL trial; ClinicalTrials.gov Identifier: NCT01862926) [49].

Another challenge for clinicians is the treatment of NM, which is often characterized by rapidly progressive proximal muscle weakness and resistance to standard immunosuppressive treatment. The use of rituximab for NM has been explored in case reports and smaller case series showing mixed results. There is evidence for beneficial effects of rituximab patients with anti-SRP-positive NM in the majority of reported cases [50–52] with a decline in CK levels, improvement in manual muscle strength and reduction of steroid doses. In anti-HMGCR-positive NM, the effects of rituximab treatment are more conflicting, with some single cases demonstrating good response or even complete remission after treatment [53, 54], while in a case series of nine patients, six were non-responders and only three showed beneficial effects including two statin-naive younger patients [55]. Evaluation of the current evidence on rituximab treatment in NM remains difficult due to the heterogeneity of patient history, treatment protocols and response parameters of the few retrospective studies on this subject. Furthermore, because of the severity of the cases, rituximab treatment was often initiated in conjunction with other medications, e.g. IVIg and cyclophosphamide, making it difficult to reliably attribute clinical improvement to rituximab alone. Larger prospective randomized trials are needed to clarify the role of rituximab in the treatment of patients with NM.

Throughout all studies in myositis, treatment with rituximab is described as generally well tolerated. A risk of serious infections, including pneumonia, urosepsis and herpes zoster, was noted in the RIM trial in which 26 serious adverse events were related to the drug amongst the 195 participants completing the trial [27]. In the register-based study by Leclair et al. three deaths resulting from severe pneumonia were reported [43]. Although rituximab is generally safe and effective in treating myositis, close treatment monitoring is necessary and patient´s comorbidities and individual risk factors need to be taken into account.

### Infliximab

The inhibition of TNF-α has been proposed as a promising therapeutic approach in experimental studies and animal models of myositis, which showed increased TNF-α expression in affected muscles and reduction of inflammation by experimental treatment with TNF-α blockers [56–58]; however, the current clinical evidence on anti-TNF-α treatment in myositis is variable. Some case reports and case series show beneficial effects [59, 60], while others report no efficacy or even exacerbation of disease after treatment with the TNF-α inhibitor infliximab (a monoclonal antibody) or etanercept (a recombinant fusion protein) [61, 62] in patients with PM and DM.

In a small randomized, double-blind, placebo-controlled trial enrolling 12 patients with refractory PM and DM, only one out of six patients receiving 5 mg/kg infliximab in the first phase met the responder criteria and another three patients improved in the second phase of the trial, in which non-responders were increased to 7.5 mg/kg infliximab and all six patients in the placebo arm received 5 mg/kg infliximab [63]. On the whole, the trial was deemed negative, although some patients showed a positive response to infliximab.

Remarkably, there are reports in the literature on new onset of DM, PM or ASS after initiation of anti-TNF-α treatment in patients affected by other diseases, e.g. rheumatoid arthritis (reviewed in [64]), although it remains unclear whether TNF-α-inhibition triggered a general immunological adverse reaction or specifically induced an unwanted inflammation in the skeletal muscle. More basic and clinical research on such mechanisms is needed in order to understand these complex immunological phenomena associated with targeted biological therapies.

### Tocilizumab

There is evidence for a potential role of IL-6 in the pathogenesis of myositis, and experimental mouse models suggest that IL-6 blockade alleviates the inflammatory response in muscle [65, 66]. Tocilizumab blocks the effect of IL-6, and its beneficial effects are known in the treatment of other autoimmune disorders, e.g. rheumatoid arthritis. There have been few anecdotal reports and case series on the successful use of tocilizumab in refractory PM, DM and ASS [67–70]. An open-label pilot study has explored the effects of tocilizumab in 11 patients with refractory NM, including 3 anti-HMGCR- and 8 anti-SRP-positive patients [71]. After 6 months of treatment, 7 patients showed a significant improvement according to the 2016 Total Improvement Score (TIS) criteria, which also correlated with baseline serum IL-6 levels and the percentage of CD56-positive muscle fibres in muscle biopsy.

Recently, a randomized, double-blind, controlled phase IIb trial evaluating the efficacy of tocilizumab in patients with refractory DM and PM has been completed (ClinicalTrials.gov Identifier: NCT02043548) and the data were presented at the ACR conference in 2020 [72]. In this study, 36 adult patients were randomized to receive either active drug or placebo for 6 months. No significant difference in the primary endpoint (TIS) could be observed over 24 weeks when comparing the groups of tocilizumab and placebo in the 32 subjects that completed the trial. However, there was a significant improvement of the TIS in both treatment arms. Publication of the final analysis needs to be awaited in order to fully assess the validity of the study for the evaluation of tocilizumab in myositis.

### Sifalimumab

Interferon (IFN) pathways are closely connected with the pathogenesis of JDM, DM and PM and seem to be one of the key players in myositis [73]. Type 1 interferons (IFN-1) include IFNα and IFNβ, and are associated with the upregulation of multiple pro-inflammatory genes in patients with DM [74–76] and PM [75].

Sifalimumab, a human monoclonal antibody targeting interferon α, was examined in a double-blind, phase 1b, multicenter, randomized, controlled trial involving 25 DM and 26 PM patients. Participants received the drug over a period of 6 months at different dosings. The study evaluated pharmacodynamic markers in blood and muscle of treated patients as well as the tolerability and safety of sifalimumab [77]. The outcome measure was the suppression of the IFN gene signature in blood and muscle at day 98 of treatment in comparison to the baseline examination and was significantly reduced in two of the three dosing groups. The modulation of the IFN gene signature positively correlates with clinical improvements on manual muscle testing.

A phase II open-label study with sifalimumab in patients with systemic lupus erythematosus and myositis, evaluating long-term safety, was completed in 2016. From 103 participants, only 67 patients completed the study as nearly every patient had an adverse event and 27.8% even experienced a serious adverse event. Although the observations were submitted to ClinicalTrials.gov, final results have not been published yet and the development of sifalimumab was discontinued in favour of another type 1 IFN inhibitor (ClinicalTrials.gov Identifier: NCT00979654).

### Belimumab

B-cell-activating factor of the tumour necrosis factor family (BAFF, also known as BLyS) is an important factor for B-cell maturation, and preclinical studies have shown elevated serum levels of BAFF in patients with myositis [78]. Belimumab is targeted against BAFF and has been approved for the treatment of systemic lupus erythematosus (SLE). Recently, results from a randomized, double-blind, controlled trial on the efficacy and safety of belimumab in myositis (ClinicalTrials.gov Identifier: NCT02347891) were presented at the ACR conference in 2021 [79]. Sixteen patients with refractory PM or DM were treated for 40 weeks. Of the belimumab group, 37.5% reached the primary endpoint-defined improvement, and in the following 24-week open-label extension phase, 42.9% of patients initially on belimumab achieved defined improvement. Nevertheless, the differences compared with the placebo arm were not statistically significant.

### Basiliximab

Basiliximab blocks the IL-2 receptor, which is expressed on activated T-cells and B-cells. There is evidence for correlation of IL-2 receptor levels with disease activity in patients with active DM [80]. In a case series of four adult patients with rapidly progressive ILD associated with anti-MDA5-positive DM, three of four patients showed a beneficial response to treatment with basiliximab [81].

Currently, a 52-week, open-label trial on the safety and efficacy of basiliximab as an add-on treatment for patients with interstitial pneumonia in amyopathic dermatomyositis (CADM) is enrolling (ClinicalTrials.gov Identifier: NCT03192657); the primary outcome measure is survival at 52 weeks.

### Antibody Therapy Targeting Complement Activation

Microvascular deposition of complement and formation of the C5-9 membrane attack complex have been shown to play a pivotal role in the pathogenesis of DM [82]. Eculizumab is directed at the complement component C5 and inhibits the cleavage of C5 to its components (C5a and C5b) and thus the formation of the membrane attack complex (MAC). There is a case report on the successful use of complement inhibition in a 19-year-old patient with severe, life-threatening DM, which was resistant to steroids, IVIg and plasma exchange, but significantly improved after induction of eculizumab [83]. A randomized, placebo-controlled, phase II trial evaluating the safety and efficacy of eculizumab in DM patients had been initiated, but the results have not been reported to date (ClinicalTrials.gov Identifier: NCT00005571).

Complement activation and MAC deposition have been suggested to be also involved in muscle fibre necrosis and inflammation in NM [84]. Furthermore, there has been evidence for a protective role of C3 deficiency in a mouse model of NM, suggesting a complement-targeting therapy in NM [85]. Despite the promising experimental findings, a phase II, randomized, double-blind, placebo-controlled clinical trial of zilucoplan, a subcutaneous peptide inhibitor of C5, showed no significant clinical effects in patients with NM and was therefore prematurely terminated (ClinicalTrials.gov Identifier: NCT04025632).

IVIg exerts part of its anti-inflammatory effects through inhibition of the complement pathway; studies in patients with DM demonstrate that IVIg inhibits the uptake of C3b and thus prevents the formation and capillary deposition of MAC [17, 86, 87]. This mechanism might also explain the effectiveness of IVIg in other neurological diseases associated with complement activation, e.g. Guillain–Barré syndrome and myasthenia gravis [88].

## Antibody Therapies in IBM

As mentioned above, the pathophysiology of IBM differs from that of the other myositis subgroups. In addition to inflammatory mechanisms, degeneration is a crucial factor for muscle damage in IBM. The majority of available data suggests that inflammation comes first and can trigger degeneration in the affected muscle cells.

A few antibodies with very different targets have been studied in IBM.

### Bimagrumab

The myostatin/activating type II receptor pathway is an important factor for the control of muscle mass. Myostatin was identified as a negative regulator of skeletal muscle mass, while primarily binding the activin type IIB receptor (ActRIIB) [89]. For preventing receptor binding of myostatin and inhibiting the negative regulation of muscle hypertrophy and muscle differentiation, a human anti-ActRII antibody, called bimagrumab, has been developed [90]*.*

To investigate the effect of bimagrumab in patients with IBM, 14 patients were treated with a single intravenous infusion in a randomized, placebo-controlled, double-blind, parallel-arm, proof-of-concept study. Eight weeks after the infusion, treated patients showed a significant increase in thigh muscle mass and lean body mass [91].

The promising results led to a multicenter, double-blind, placebo-controlled phase IIb study (“RESILIENT trial”), in which 251 participating patients were randomized into 3 groups with different dosages of bimagrumab and one placebo group over a treatment period of 48 weeks. However, the primary outcome, the 6-min walking distance (6MWD), did not differ between the bimagrumab groups and the placebo group at week 52 [28].

The extended treatment period up to 2 years with bimagrumab was well tolerated, but could not reach improvement in mobility [92]. Patients of the extension study showed an increase of lean body mass, but without any resulting clinical improvement [93].

### Alemtuzumab

Alemtuzumab, a humanized anti-CD52 monoclonal antibody, depletes B-cells and T-cells. The drug is licensed for severe courses of relapsing–remitting multiple sclerosis [94].

The effect of one series of alemtuzumab infusion in IBM was examined in a proof-of-concept study with 13 patients (“CAMPATH 1-H trial”). All patients had an established and documented 12-month natural history of disease progression. The patients received 0.3 mg/kg/day alemtuzumab for 4 days. Compared to the historical natural disease course of the patients, the decline of muscle strength was halted 6 months after the infusion and six patients displayed an improvement in the performance of daily activities. All patients underwent muscle biopsy at baseline, before the infusion and 6 months after the treatment. Six months after treatment, endomysial inflammation and stressor molecules were reduced in muscle biopsy [95]. However, further work-up of the muscle biopsies from treated patients could not demonstrate an effect on the most important markers of degeneration and cell stress, including amyloid precursor protein, αB-crystallin and ubiquitin [96]. A more recent case report on a IBM patient treated with alemtuzumab reported disease stabilization in a follow-up of 2 years [97]. Nevertheless, no larger trials of alemtuzumab in IBM have been initiated so far.

In a case report of a patient with refractory polymyositis, treatment with alemtuzumab showed a beneficial response with regard to walking distance, CK level and prednisolone tapering [98].

### Canakinumab

The pro-inflammatory cytokine IL-1β is secreted by monocytes and macrophages. In muscle cells, IL-1β colocalizes with amyloid precursor protein and induces an overexpression of amyloid precursor protein. Consequently, accumulation of β-amyloid occurs with subsequent cell stress, resulting in cell death [99, 100]. Due to this mechanism of action, specific blocking of IL-1β appeared to be a reasonable treatment target in IBM.

Canakinumab is a monoclonal antibody against IL-1β, and the effect was investigated in a proof-of-principle, open-label study in 5 patients with IBM [101]. The patients received subcutaneous canakinumab every 8 weeks over a mean period of 15.8 months. Bimonthly grip strength and the total muscle strength were evaluated. Canakinumab was well tolerated but showed no meaningful clinical improvement in the 5 patients. Further studies with canakinumab are not yet planned.

Prior to canakinumab, inhibition of IL-1 in IBM had also been studied using anakinra, a recombinant IL-1 receptor antagonist which blocks the activity of IL-1α and IL-1β. In a small pilot study, four IBM patients received anakinra, but no improvement of muscle strength or grip strength was found. The authors had discussed whether the ineffectiveness was due to the fact that the therapy time was too short or that an intramuscular effect could not be achieved with anakinra [102]. Besides IBM, anakinra was examined in refractory DM and PM. In a mechanistic study with 15 participants, a clinical response according to the International Myositis Assessment and Clinical Studies Group (IMACS) core set measures was found in 7 patients. Treatment response was associated with changes in extramuscular score, IL-1α expression, blood CD4 activated/memory T-cells and muscle CD163 macrophages [103].

## Conclusion

Disease progression, severity and extramuscular involvement differ between individual myositis patients. Patients with myositis mostly respond well to treatment with long-term, low-dose glucocorticosteroids and an immunosuppressant like azathioprine or methotrexate. But refractory weakness, dysphagia and interstitial lung disease can be life-threatening, and more aggressive treatment options are needed. In these cases, monoclonal antibodies offer great potential for target-specific treatment.

There is considerable evidence for successful use of antibody therapy in myositis: rituximab has shown beneficial effects in patients with refractory PM, DM, ASS and associated ILD. As a humoral, immunomodulatory agent rather than a monoclonal antibody, IVIg is successfully used in the treatment of various autoimmune diseases and, upon completion of a pivotal controlled trial, the first treatment for adult DM that has been approved by the FDA and EMA.

Infliximab, sifalimumab and bimagrumab have failed expectations in randomized trials. Studies on tocilizumab, belimumab and alemtuzumab gave ambiguous results in small, limited clinical studies. Treatment of NM remains challenging and often requires combination treatment with multiple immunosuppressive agents. Because of the possibility of severe adverse events, the use of antibodies needs to be carefully considered with regard to the individual risk profile of the patient.

Besides therapeutical antibodies, biologic agents such as abatacept and JAK inhibitors also hold promise for the treatment of severe cases of myositis and are investigated in ongoing randomized phase II/III trials.

Overall, larger, well-controlled studies in myositis are still scarce. Clinical trials in myositis remain challenging due to the rarity and heterogeneity of the disease. Small sample sizes are more prone to variability regarding treatment effects, and the differentiation of the subtypes ASS, OM and NM from PM has been established only in recent years; these subtypes have distinct pathomechanisms, which might partly explain the heterogeneous responses to target-specific biologicals in clinical trials. Furthermore, varying outcome measures in clinical trials complicate interpretation of study results. Therefore, an important step towards a more comparable and reliable assessment of treatment efficacy in clinical trials is the standardization of response criteria in myositis, such as those proposed by the IMACS [104], which have already been employed in several recent trials.

In addition to optimization of trial design, ongoing research effort on disease mechanisms in myositis is essential. Especially in IBM, further understanding of the pathophysiology will hopefully lead to the identification of new targets for antibody therapy or other treatment modalities. Although there are treatment options available for DM, PM, ASS and NM, many open questions remain, such as the role of complement in NM or the complexity of pro-inflammatory and immunosuppressive functions of cytokines in DM and PM.

Future priorities for target-specific treatment in myositis are to continue to improve our understanding of disease mechanisms and to conduct randomized controlled trials using standardized outcome measures and appropriate sample sizes through multicenter collaborations. These efforts will hopefully optimize the application of targeted therapies in patients with myositis.

## Supplementary Information

Below is the link to the electronic supplementary material.

Supplementary file1 (PDF 499 KB)

Supplementary file2 (PDF 507 KB)

Supplementary file3 (PDF 499 KB)
